# Simultaneous Occurrence of Rosai–Dorfman Disease and Nodal Marginal Zone Lymphoma in a Patient with Sjögren's Syndrome

**DOI:** 10.1155/2018/7930823

**Published:** 2018-09-16

**Authors:** Vadim R. Gorodetskiy, Wolfram Klapper, Natalya A. Probatova, Vladimir I. Vasilyev, Elena V. Rozhnova

**Affiliations:** ^1^Department of Intensive Methods of Therapy, V.A. Nasonova Research Institute of Rheumatology, Kashirskoye Shosse 34A, Moscow 115522, Russia; ^2^Department of Pathology, Hematopathology Section and Lymph Node Registry, Christian-Albrecht University Kiel and University Hospital Schleswig-Holstein, 24105 Kiel, Germany; ^3^Department of Pathology, N.N. Blokhin Russian Cancer Research Center, Kashirskoye Shosse 24, Moscow 115478, Russia; ^4^Department of Therapeutic Dentistry, I.M. Sechenov First Moscow State Medical University, Trubetskaya, 8, Building 2, Moscow 119991, Russia

## Abstract

We present an exceptionally rare case of co-occurrence of Rosai–Dorfman disease (RDD) and nodal marginal zone lymphoma (NMZL) in a 60-year-old Caucasian female with a 20-year course of Sjögren's syndrome (SS). In response to treatment for lymphoma, the patient presented a short positive response, followed by a rapid progression of the disease accompanied by the development of the peripheral facial nerve palsy. We failed to detect Epstein–Barr virus (EBV) in the NMZL/RDD sample by EBV-encoded RNA (EBER) in situ hybridization but identified genomic DNA of EBV by polymerase chain reaction. A second biopsy revealed EBV-positive diffuse large B-cell lymphoma (DLBCL), not otherwise specified. The identical clonal immunoglobulin heavy chain gene rearrangements in the NMZL and DLBCL pointed to their clonal relationship. Though the role of EBV in the pathogenesis of some lymphomas is well-known, there have been only few cases of EBV-induced transformation of low-grade B-cell lymphoma into high-grade lymphoma and no cases of a patient with an NMZL background. To our knowledge, this is the first report of a concomitant occurrence of RDD and NMZL in a SS patient.

## 1. Introduction

Sjögren's syndrome (SS) is a systemic autoimmune disease characterized by chronic inflammation of the exocrine glands, mainly the salivary and lacrimal glands [1]. Among autoimmune diseases, SS is known to have the highest incidence of lymphomas [2]. The latest World Health Organization (WHO) classification distinguishes three subtypes of B-cell marginal zone lymphoma (MZL) according to the sites involved: extranodal marginal zone of mucosa-associated lymphoid tissue (MALT) lymphoma, splenic MZL (SMZL), and nodal MZL (NMZL) [3]. MZL, particularly MALT lymphoma of the salivary glands, is the most common histological type of lymphoma encountered in the setting of SS [4–6].

Rosai–Dorfman disease (RDD, sinus histiocytosis with massive lymphadenopathy) is a rare idiopathic histiocytic disorder [7]. While the pathogenesis of RDD remains elusive, the new histiocytic society has classified this disease into the following categories: familial, classical (nodal), extranodal, neoplasia-associated, and immune disease-associated [8]. Although a few hundred publications related to RDD can be found in the literature, only 28 cases of RDD associated with non-Hodgkin and Hodgkin lymphomas have been reported to date. Furthermore, the simultaneous occurrence of RDD and NMZL is extremely rare, and to the best of our knowledge, only two cases of this kind have been documented [9, 10]. The association of RDD with autoimmune diseases has also been reported; however, we were able to find only two documented cases describing the co-occurrence of SS and RDD [11, 12]. This report presents an exceptionally rare case of simultaneous occurrence of RDD and NMZL in a female with SS.

## 2. Case Presentation

In February 2013, a 60-year-old Caucasian woman was admitted to the V. A. Nasonova Research Institute of Rheumatology with complaints of weakness, weight loss of 25 kg over 2 years, dryness of the eyes and mouth, and enlargement of the cervical and axillary lymph nodes. Her medical history was consistent with a 20-year course of SS. Since May 2011, she had experienced numbness in her feet followed by the development of Raynaud's syndrome and recurrent purpura on the shins. Increasing and decreasing lymphadenopathy involving the neck and axillae was observed over 2 years. Within 3 months before admission, she was taking 4 mg of methylprednisolone every other day.

At the time of admission, peripheral blood counts were as follows: hemoglobin 13.6 g/dl, platelets 224 × 10^9^/L, and white blood cells 6.7 × 10^9^/L, with 85% neutrophils, 8% lymphocytes, and 7% of monocytes. Laboratory data showed that electrolytes and renal and liver function were within the normal range. The serum lactate dehydrogenase level was elevated to 593 IU/L (normal **<**378). Ophthalmic examination revealed that the patient had keratoconjunctivitis sicca (with filamentous keratitis), with a Schirmer's test score of <1 mm/5 min and tear break-up time of 3-4 sec (OD) and 8 sec (OS). Dental examination of the submandibular and parotid salivary glands showed no salivation (sialometry: 0 mL). A labial salivary gland biopsy showed marked focal lymphocytic sialadenitis with focus score of 4 foci per 4 mm^2^. Anti-SSA/Ro antibody level exceeded 200 U/ml (normal < 25), antinuclear antibody level was 1:640 (normal < 1:160) with homogeneous and speckled patterns, and rheumatoid factor level was 885 IU/mL (normal < 20). Based on the clinical, serological, and pathological features, the diagnosis of SS was confirmed.

Protein electrophoresis and immunofixation of the patient's serum showed monoclonal immunoglobulin (Ig) M kappa (1.5 g/L) and a decrease in polyclonal IgG and IgA levels. The monoclonal Ig had the property of cryoglobulin. Urine protein electrophoresis and immunofixation assays detected the presence of monoclonal free kappa type light chains (0.06 g/24 h). Increased *β*2-microglobulin and C-reactive protein levels were noted to be as high as 5.99 mg/L (normal < 2.4) and 47.7 mg/L (normal < 6.0), respectively. In addition, the C4 complement level was reduced to 0.02 g/L (normal range 0.1–0.4). A full staging computerized tomography (CT) scan showed multiple enlarged lymph nodes in the neck, axillary, mediastinal, lung hilar, abdominal, and retroperitoneal spaces, with a fusion of the conglomerates (maximum size of 4.1 × 2.4 cm) and mild splenomegaly (measuring 8.2 × 13.3 × 15.2 cm). Additionally, bilateral lymphangitis was found in the lung parenchyma, and the patient had developed moderate bilateral hydrothorax with compression of basal segments of the lower lobes.

**Figure 1:**
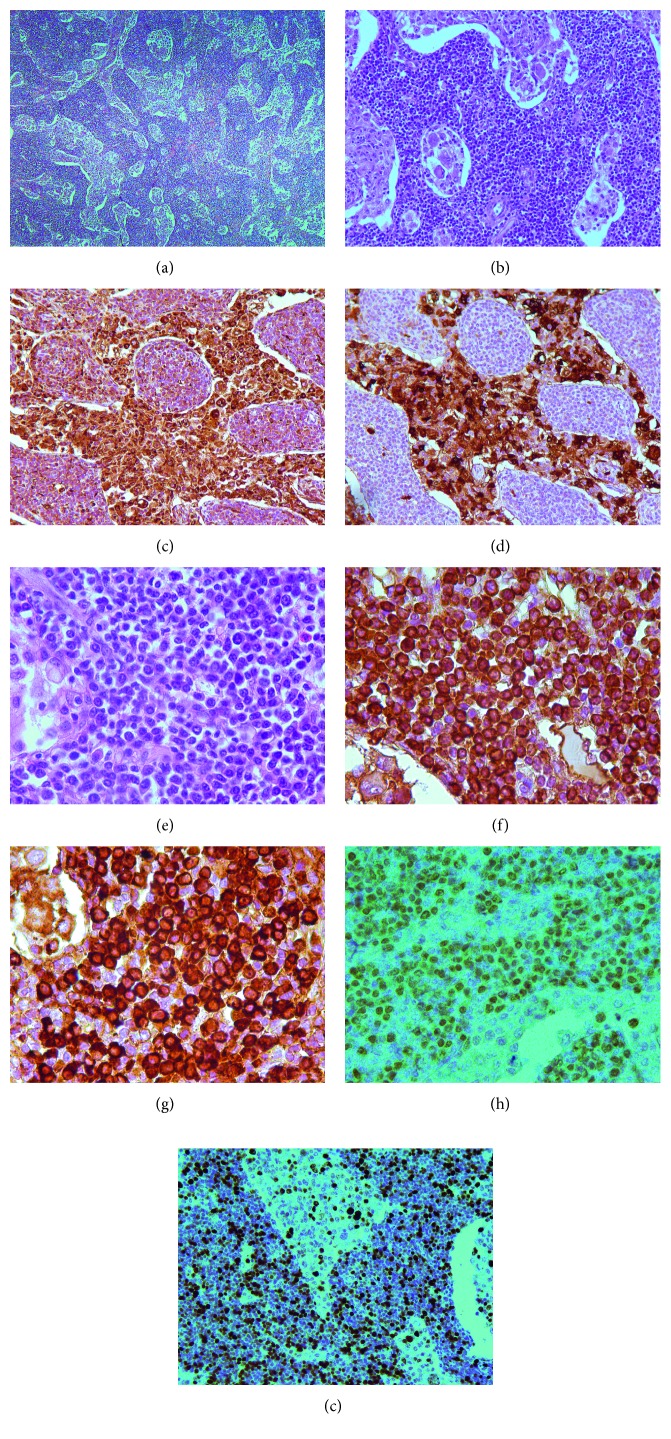
(a) The lymph node shows a completely effaced architecture. The sinus system is open and filled with partly pleomorphic histiocytic cells (×50, hematoxylin and eosin stain (H&E)). (b) The partly pleomorphic histiocytic cells are present in the sinuses, while the intervening cords exhibit a marked plasmacytosis (×200, H&E). (c) The histiocytic cells express CD68 (×200). (d) The histiocytic cells show nuclear and cytoplasmic positivity for S100 (×200). (e) The mature plasma cells (Marschalko type) present in the intervening cords (×630, H&E). (f) Monomorphic expression of light kappa chain in plasma cells (×630). (g) Monomorphic expression of IgM in plasma cells (×630). (h) Expression of MUM1 in plasma cells (×630). (i) Ki-67 is expressed in 5–7% of the cells of the sinuses and up to 50% of cells within the intervening cords of the lymph node (×200).

**Figure 2:**
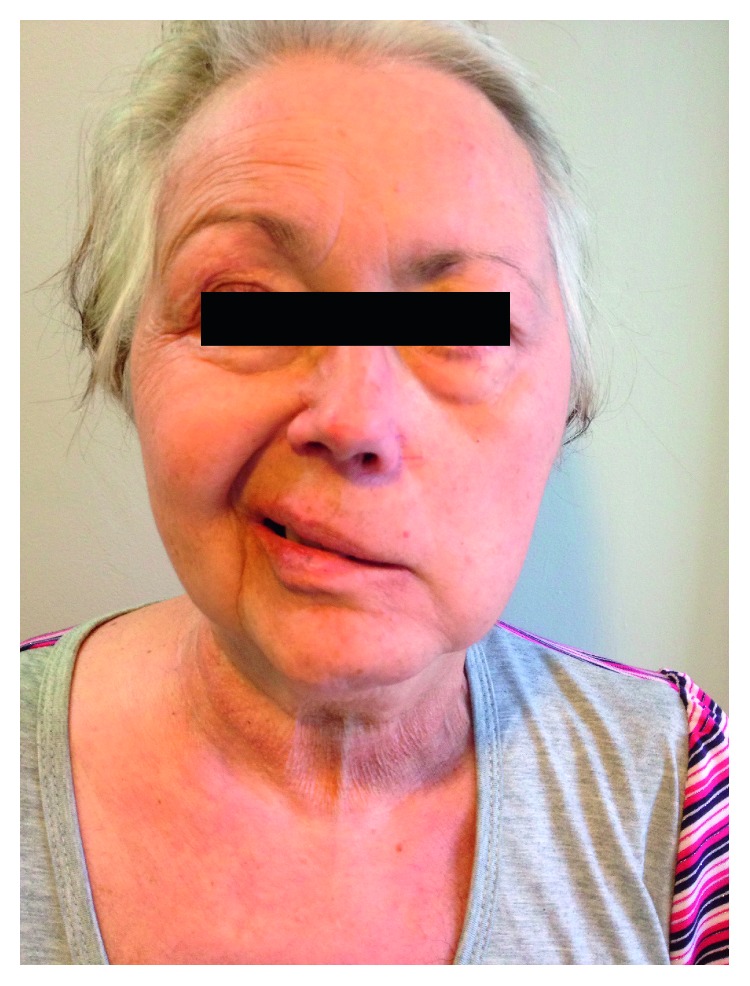
Left-sided peripheral facial nerve palsy with an inability to wrinkle the forehead and nose, unequal lid fissures, and inability to lift the corner of the mouth.

**Figure 3:**
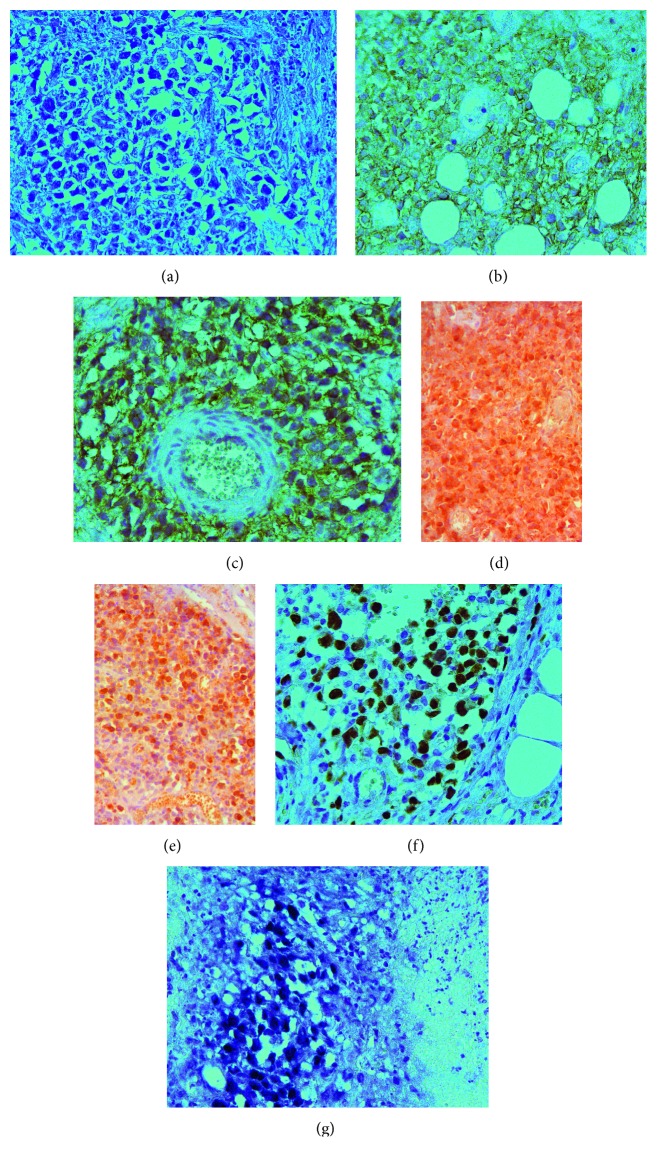
(a) The focus of large lymphoid cells, surrounded by necrotic tissue (×400, H&E). B–F. Immunostaining of the large tumor cells expressing CD20. (b) (×400), CD30 (c) (×400), light kappa chain. (d) (×200), IgM. (e) (×200), MUM1. (f) (×400). (g) Detection of EBV-positive large lymphocytes using EBER in situ hybridization.

Histologic examination of the right axillary lymph node revealed a completely effaced architecture (Figure 1(a)). The sinus system was open and filled with pleomorphic histiocytic cells, some of which showed emperipolesis of peripheral blood cells (Figure 1(b)). Immunohistochemical staining showed the expression of CD68 (Figure 1(c)) and CD163 in these histiocytic cells accompanied by coexpression of S100 (Figure 1(d)), while the staining result was negative for CD1a and Langerin. The lymph node tissue showed an infiltrate consisting of partly nodular and partly diffused pattern of CD20-positive and CD10-negative B-lymphocytes and extensive plasma cell infiltration (Figure 1(e)). The plasma cells showed positive staining for light kappa chain (Figure 1(f)), IgM heavy chain (Figure 1(g)), and MUM1 (Figure 1(h)). However, they did not stain positive for the expression of CD138, VS38c, CD56, and Cyclin-D1. Ki-67, a marker of cell proliferation, was expressed in 5–7% of the cells of the sinuses and up to 50% of the cells within the intervening cords of the lymph node (Figure 1(i)). We failed to detect Human herpesvirus-8 by immunohistochemistry and EBV-encoded RNA (EBER) in situ hybridization (EBER-ISH), but the DNA of the EBV was detected in the sample by the polymerase chain reaction (PCR). Further, sequence analysis of lymph node tissue showed no mutations within the codon 265 of the *MYD88* gene and somatic mutations in *KRAS* and *MAP2K1*. Histological and cytological analysis revealed no infiltration of bone marrow by lymphocytes and plasma cells. Thus, the patient was diagnosed with concomitant RDD and plasmacytic marginal zone lymphoma. After six cycles of MDB (melphalan [Alkeran] + dexamethasone + bortezomib), a partial response was achieved according to the data of the CT scan. As follow-up analyses, electrophoresis and immunofixation of serum proteins were performed that failed to detect any presence of monoclonal IgM kappa. Electrophoresis followed by immunofixation of concentrated urine proteins showed secretion of the monoclonal free kappa type light chains in trace amounts. In November 2013, a month after the end of chemotherapy, a fast-growing firm mass developed in the left mandibular fossa spreading anteriorly to the parotid area. Soon, peripheral paralysis of the facial nerve occurred (Figure 2). Histological examination of this mass revealed a necrotic tissue interspersed with foci of small lymphoid cells with scarce large tumor cells, mainly with round and oval nuclei containing nucleoli and pyroninophilic cytoplasm. Large tumor cells were also present as perivascular and perineural clusters (Figure 3(a)). Small lymphoid cells were predominantly T lymphocytes (CD3+) and expressed either CD4 or CD8, approximately in equal proportions. Large lymphoid cells expressed CD20 (Figure 3(b)), CD79α, CD30 (Figure 3(c)), light kappa chain (Figure 3(d)), IgM heavy chain (Figure 3(e)), PAX5, and MUM1 (Figure 3(f)). Expression of Ki-67 was observed in nearly 60% of tumor cells. The EBER-ISH demonstrated the presence of EBV-positive large lymphocytes (Figure 3(g)). EBV-positive diffuse large B-cell lymphoma, not otherwise specified (DLBCL, NOS) was diagnosed.

**Figure 4:**
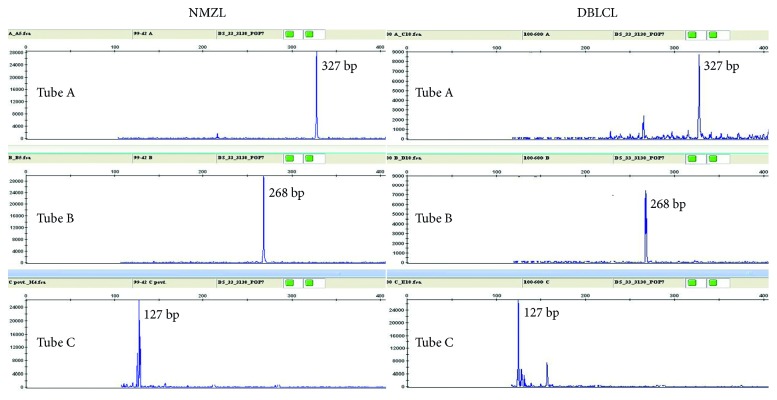
B-cell clonality analysis with IGHV FR1-IGHJ (Tube A), IGHV FR2-IGHJ (Tube B), and IGHV FR3-IGHJ (Tube C). PCR products demonstrate an identical clonal pattern between the NMZL and DLBCL.

Multiplex BIOMED-2 PCR fragment assays were used to study rearrangements of the Ig heavy chain (IGH) gene in NMZL and DLBCL samples. IGH framework 1, 2, and 3 assays (Tube A, Tube B, and Tube C) were used to detect Vh-Jh rearrangements. Identical clonal patterns between the NMZL and DLBCL were demonstrated (Figure 4). The resulting condition was considered a transformation of NMZL into an EBV-positive DLBCL, NOS. R-CHOP (rituximab, cyclophosphamide, doxorubicin, vincristine, and prednisone) immunochemotherapy was prescribed to the patient. Unfortunately, a few days following the completion of the first cycle, the patient suddenly lost consciousness and died. No postmortem study was performed.

## 3. Discussion

In the current study, the patient experienced SS for a long time and had SS-associated lymphoma risk factors, such as purpura, low levels of complement component C4, and cryoglobulinemia. Clinical data suggested the development of lymphoma, while RDD was an accidental finding. In our patient, the prominent sinuses were occupied with large histiocytes exhibiting emperipolesis and expressing CD68 and CD163 with coexpression of S100, remaining negative for CD1a and Langerin; these characteristics allowed for the diagnosis of RDD. We believe that the lymphoplasmacytic infiltrate that was composed of sheets of plasma cells within the intervening cords could be erroneously attributed to RDD. However, monotypic expression of IgM kappa by plasma cells indicated their neoplastic nature, and this finding was consistent with the presence of monoclonal IgM kappa in the serum. The admixed B-lymphocytes, which do not form physiological B-cell follicles, allowed us to classify this lymphoma as MZL with extensive plasmacytic differentiation. The lack of MYD88 mutation and any signs of bone marrow involvement argued against a diagnosis of lymphoplasmacytic lymphoma. Further, our patient showed an absence of the enlarged salivary glands, minimal enlargement of the spleen, generalized lymphadenopathy, and the lack of bone marrow involvement, confirming the diagnosis of NMZL. A less favorable diagnosis was the MALT lymphoma of the salivary gland with a secondary lymph node involvement or SMZL.

The phenomenon of the lack of CD138 expression in malignant plasma cells of MZL with plasmacytic differentiation has been described previously [13, 14]. In our case, morphologically mature malignant plasma cells (Marschalko type) in the intervening cords also exhibited an aberrant plasma cell-related antigen profile. They did not express CD138 and VS38c, recognized markers for plasma cells. Some studies have shown that the CD138-negative plasma cells form a distinct subpopulation of malignant plasma cells, possibly representing more primitive and highly proliferative cells than CD138-positive cells [15–18]. These data can explain the high index of proliferative activity that was observed in MZL cells of our patient.

In 10% of patients with RDD, an immunological disease is diagnosed [9]. However, to our best knowledge, the co-occurrence of SS and RDD has been described only in two cases. Drosos et al. described a 55-year-old man with primary SS who developed RDD after 6 years of being diagnosed with SS [11]. Another report by Maia et al. presented the case of a 40-year-old female with SS diagnosed 8 months following RDD diagnosis [12]. In both these cases, the diagnosis of RDD was established by lymph node biopsy. In our case, RDD was diagnosed in a 60-year-old woman 20 years after the SS diagnosis. The possibility of whether the association between SS and RDD is a coincidence or has a pathogenetic relationship remains to be determined.

After reviewing all the relevant literature, we found 28 case reports of RDD in association with both non-Hodgkin and Hodgkin lymphoma, but none of them had concomitant SS. Only two cases of simultaneous occurrence of RDD and NMZL have been documented. Pang et al. described a case of an 80-year-old woman with subcutaneous lymph node of the arm, and Akria at al. studied a 50-year-old man with abdominal lymphadenopathy who was also diagnosed with autoimmune hemolytic anemia [9, 10]. In both cases, as observed with our patient, RDD and NMZL were localized in the same lymph node. Given the rarity of association between RDD and lymphoma as well as the lack of evidence for clonality in RDD, it may be suggested that the RDD represents an unusual histiocytic response to lymphoma or RDD, and lymphoma arises independently in response to a common, unknown till now, etiological agent.

Multiplex PCR fragment assays showed identical clonal patterns between the NMZL and DLBCL, indicating clonal identity between the two lymphomas. However, such identical clonal relationship does not necessarily indicate a linear disease progression [19, 20]. It is possible to hypothesize that DLBCL was not a consequence of the transformation of NMZL and that both lymphomas in our case originated from a common precursor cell population. However, the expression of the same IgM kappa by tumor cells of both lymphomas suggests that DLBCL was a consequence of the clonal evolution of NMZL.

Evidence has shown that samples identified as EBV negative by immunohistochemistry and EBER-ISH demonstrated the presence of EBV-microRNAs and EBV genome [21]. In our case, we also failed to detect EBER-ISH in the lymph node affected by MZL and RDD, and only the PCR showed the presence of DNA EBV in this sample. However, tumor lymphocytes of DLBCL were EBV-positive. It can be assumed that after chemotherapy, the reactivation of EBV occurred, which became the trigger responsible for the transformation of NMZL into DLBCL. Although an association between EBV infection and lymphoma development is well-established, very few cases of EBV-induced transformation of the low-grade B-cell lymphoma into high-grade lymphoma have been described [22–29]. To our knowledge, this is the first documented case of EBV-induced transformation of the NMZL into DLBCL.

In conclusion, the current study describes an exceptionally rare case of simultaneous occurrence of RDD and NMZL in a patient with SS. We further believe that in the presented case, reactivation of a latent EBV infection by immunosuppressive therapy within a preexisting NMZL led to the development of the secondary EBV-positive DLBCL, NOS.

## References

[1] Fox R. I., Howell F. V., Bone R. C., Michelson P. E. (1984). Primary Sjogren syndrome: clinical and immunopathologic features. *Seminars in Arthritis and Rheumatism*.

[2] Zintzaras E., Voulgarelis M., Moutsopoulos H. M. (2005). The risk of lymphoma development in autoimmune diseases: a meta-analysis. *Archives of Internal Medicine*.

[3] Swerdlow S. H., Campo E., Harris N. L. (2017). *WHO Classification of Tumours of Haematopoietic and Lymphoid Tissues*.

[4] Voulgarelis M., Dafni U. G., Isenberg D. A. (1999). Malignant lymphoma in primary Sjogren’s syndrome: a multicenter, retrospective, clinical study by the European concerted action on sjogren’s syndrome. *Arthritis & Rheumatism*.

[5] Tzioufas A. G. (1996). B-cell lymphoproliferation in primary sjogren’s syndrome. *Clinical and Experimental Rheumatology*.

[6] Royer B., Cazals-Hatem D., Sibilia J. (1997). Lymphomas in patients with Sjogren’s syndrome are marginal zone B-cell neoplasms, arise in diverse extranodal and nodal sites, and are not associated with viruses. *Blood*.

[7] Foucar E., Rosai J., Dorfman R. F. (1990). Sinus histiocytosis with massive lymphadenopathy (Rosai-Dorfman disease): review of the entity. *Seminars in Diagnostic Pathology*.

[8] Emile J. F., Alba O., Fraitag S. (2016). Revised classification of histiocytoses and neoplasms of the macrophage-dendritic cell lineages. *Blood*.

[9] Pang C. S., Grier D. D., Beaty M. W. (2011). Concomitant occurrence of sinus histiocytosis with massive lymphadenopathy and nodal marginal zone lymphoma. *Archives of Pathology & Laboratory Medicine*.

[10] Akria L., Sonkin V., Braester A., Cohen H. I., Suriu C., Polliack A. (2013). Rare coexistence of Rosai-Dorfman disease and nodal marginal zone lymphoma complicated by severe life-threatening autoimmune hemolytic anemia. *Leukemia & Lymphoma*.

[11] Drosos A. A., Georgiadis A. N., Metafratzi Z. M., Voulgari P. V., Efremidis S. C., Bai M. (2004). Sinus histocytosis with massive lymphadenopathy (Rosai-Dorfman disease) in a patient with primary sjogren’s syndrome. *Scandinavian Journal of Rheumatology*.

[12] Maia R. C., de Meis E., Romano S., Dobbin J. A., Klumb C. E. (2015). Rosai-Dorfman disease: a report of eight cases in a tertiary care center and a review of the literature. *Brazilian Journal of Medical and Biological Research*.

[13] Coupland S. E., Hellmich M., Auw-Haedrich C., Lee W. R., Anagnostopoulos I., Stein H. (2005). Plasmacellular differentiation in extranodal marginal zone B cell lymphomas of the ocular adnexa: an analysis of the neoplastic plasma cell phenotype and its prognostic significance in 136 cases. *British Journal of Ophthalmology*.

[14] Meyerson H. J., Bailey J., Miedler J., Olobatuyi F. (2011). Marginal zone B cell lymphomas with extensive plasmacytic differentiation are neoplasms of precursor plasma cells. *Cytometry Part B: Clinical Cytometry*.

[15] Joshua D., Peterson A., Brown R., Pope B., Snowdon L., Gibson J. (1996). The labelling index of primitive plasma cells determines the clinical behaviour of patients with myelomatosis. *British Journal of Haematology*.

[16] Pope B., Brown R., Gibson J., Yuen E., Joshua D. (2000). B7-2-positive myeloma:incidence, clinical characteristics, prognostic significance and implications for tumour therapy. *Blood*.

[17] Matsui W., Huff C., Wang Q. (2004). Characterisation of clonogenic multiple myeloma cells. *Blood*.

[18] Reid S., Yang S., Brown R. (2010). Characterisation and relevance of CD138-negative plasma cells in plasma cell myeloma. *International Journal of Laboratory Hematology*.

[19] Fitzgibbon J., Iqbal S., Davies A. (2007). Genome-wide detection of recurring sites of uniparental disomy in follicular and transformed follicular lymphoma. *Leukemia*.

[20] Liu H., Yan Q., Nuako-Bandoh B. (2012). Richter transformation: clonal identity does not indicate a linear disease progression. *British Journal of Haematology*.

[21] Mundo L., Ambrosio M. R., Picciolini M. (2017). Unveiling another missing piece in EBV-driven lymphomagenesis: EBV-encoded microRNAs expression in EBER-negative Burkitt lymphoma cases. *Frontiers in Microbiology*.

[22] Strunk J. E., Schuttler C, Ziebuhr J (2013). Epstein-barr virus–induced secondary high-grade transformation of sjogren’s syndrome–related mucosa-associated lymphoid tissue lymphoma. *Journal of Clinical Oncology*.

[23] Terasawa T., Ohashi H., Utsumi M. (2003). Case of epstein-barr virus-associated transformation of mantle cell lymphoma. *American Journal of Hematology*.

[24] Tao J., Kahn L. (2000). Epstein-Barr virus–associated high-grade B-cell lymphoma of mucosal-associated lymphoid tissue in a 9-year-old boy. *Archives of Pathology & Laboratory Medicine*.

[25] Rubin D., Hudnall S. D., Aisenberg A., Jacobson J. O., Harris N. L. (1994). Richter’s transformation of chronic lymphocytic leukemia with Hodgkin’s-like cells is associated with Epstein-Barr virus infection. *Modern Pathology*.

[26] Petrella T., Yaziji N., Collin F. (1997). Implication of the epstein-barr virus in the progression of chronic lymphocytic leukaemia/small lymphocytic lymphoma to hodgkin-like lymphomas. *Anticancer Research*.

[27] Menon M. P., Hutchinson L., Garver J., Jaffe E. S., Woda B. A. (2013). Transformation of follicular lymphoma to epstein-barr virus–related hodgkin-like lymphoma. *Journal of Clinical Oncology*.

[28] Ansell S. M., Li C. Y., Lloyd R. V., Phyliky R. L. (1999). Epstein-Barr virus infection in Richter’s transformation. *American Journal of Hematology*.

[29] Ambrosio M. R., Falco G. D., Gozzetti A. (2014). Plasmablastic transformation of a pre-existing plasmacytoma: a possible role for reactivation of epstein barr virus infection. *Haematologica*.

